# Identifying symptom recurrences in primary brain tumor patients using the MDASI-BT and qualitative interviews

**DOI:** 10.1186/s41687-019-0143-0

**Published:** 2019-08-23

**Authors:** Alvina A. Acquaye, Samuel S. Payén, Elizabeth Vera, Loretta A. Williams, Mark R. Gilbert, Shiao-Pei Weathers, Terri S. Armstrong

**Affiliations:** 1 Neuro-Oncology Branch, National Institutes of Health, 9030 Old Georgetown Rd, Room 231, Bethesda, MD 20892 USA; 2 Department of Family Health Houston, The University of Texas Health Science Center of Nursing Research, Houston, TX USA; 3 Department of Symptom Research, Anderson Cancer Center, Houston, TX USA; 4 Department of Neuro-Oncology, Anderson Cancer Center, Houston, TX USA

**Keywords:** MDASI-BT, Patient outcomes, Brain tumors, Quality of life, Symptom management, Qualitative research

## Abstract

**Background:**

Identifying symptoms experienced throughout the disease trajectory is pivotal to understanding management of patient symptoms. Patient interviews to solicit input from those who have experienced these symptoms is one method to capture this perspective to validate symptoms included in patient reported outcomes (PRO) measures.

**Methods:**

A thematic approach was used to identify themes within qualitative interviews. The MD Anderson Symptom Inventory- Brain Tumor (MDASI-BT) was completed by glioma patients. Descriptive statistics was used for analysis of the MDASI-BT.

**Results:**

Thematic saturation was reached with 23 participants, with a median age of 53 (23–62), on treatment (57%) and diagnosed with a glioblastoma (48%). Patients endorsed 20 out of the 22 MDASI-BT symptoms (symptoms not reported: dry mouth, shortness of breath) during the interviews and with completion of the instrument (seizures and vomiting were not endorsed). Fatigue (55%), seizures (50%), and pain (50%) were common symptoms described by the sample. During treatment, more symptoms were identified with fatigue, hair loss, and nausea more problematic. Aside from itching and swelling (endorsed by 2 patients each), all other symptoms not included in the MDASI-BT instrument were endorsed by only one patient.

**Conclusions:**

Completion of the MDASI-BT, found patients reported on average 6.8 symptoms with 14% of reported symptoms (mean = 3) rated as moderate to severe. The findings demonstrate how applicable the MDASI-BT is in capturing significant symptoms experienced and how important it is to utilize throughout ones’ care to manage symptoms effectively.

**Electronic supplementary material:**

The online version of this article (10.1186/s41687-019-0143-0) contains supplementary material, which is available to authorized users.

## Background

There are over 100 distinct types of primary brain and central nervous system (CNS) tumors with nearly 80,000 new cases expected to be diagnosed in 2018 [1]. Tumors can arise from the meninges or within the brain parenchyma [1]. Tumors can be considered either low grade or malignant, with malignant tumors accounting for nearly one-third of primary brain and CNS tumors [1]. Clinical presentation is variable; patients often presenting with neurologic or cognitive symptoms based on lesion size and location [2]. Patients may develop additional symptoms associated with concomitant medications, treatment, and comorbidities [3].

Gliomas are the most common malignant primary brain tumor and providing care for patients is complex. Studies have demonstrated high symptom burden, a high number of concurrent and severe symptoms, including those emanating from the location within the brain in addition to more generalized symptoms [3, 4]. The use of a routine screening tool for common symptoms may promote improved symptom management, as has recently been demonstrated to improve survival in patients with other solid tumor malignancies [5].

The field of neuro-oncology is continuing to evolve as evidenced by the development of new approaches to surgery, radiation, and systemic treatments, such as chemotherapy. However, recognition of existing limitations in understanding the clinical benefit of treatment on how the patient feels and functions led to a workshop sponsored by the Jumpstarting Brain Tumor Drug Development Coalition (National Brain Tumor Society, Society for Neuro-Oncology, Musella Foundation for Brain Tumor Research and Information, and Accelerate Brain Cancer Cure), to evaluate ways to include this evaluation as part of clinical trials in patients with a malignant glioma [4, 6]. The Jumpstart Brain Tumor Drug Development Coalition and U.S. Food and Drug Administration (FDA) Clinical Trials Clinical Outcome Assessment (COA) Endpoints Workshop held in 2014, included participation from the FDA and stakeholders from the brain tumor community, including researchers, clinicians, industry, patients, patient advocates, and clinical research organizations and included discussion of the core principles of current COA tools to further enhance measurements for prospective clinical trials [6, 7]. To gain understanding of symptoms experienced through the disease trajectory, workshop planning members had brain tumor patients and caregivers complete an online survey primarily focused on signs, symptoms, and functions essential to them for addition into clinical trials [6]. Results showed common presenting symptoms endorsed by high grade glioma patients included headaches, seizures, and changes in speech and mood [6]. Findings from the workshop encouraged the inclusion of COAs in clinical trials, with an emphasis on assessing symptoms and developing measures to evaluate function.

Various types of COAs exist, with patient-reported outcome (PRO) measures focused on the patients’ perspective of their symptoms [7]. The FDA describes PRO instruments as “measurements of any aspect of a patient’s health status that comes directly from the patient without the interpretation of the patient’s response by a physician or anyone else [8]”. The MD Anderson Symptom Inventory-Brain Tumor (MDASI-BT) is an instrument developed specifically for use in glioma patients. Building on the core MDASI, symptoms specific to this patient population were identified from the literature and content validity was assessed by a panel of expert health care professionals, patients, and family members [9]. The MDASI-BT is a measure of symptom burden that consists of 22 symptoms and 6 interference items of daily life. The MDASI-BT has demonstrated psychometric validity and reliability [9, 10]. Symptom burden is the combined impact of disease and treatment symptoms on patient functioning [11].

The FDA published guidance on development of PROs, states that “Item generation should include input from the target patient population to establish the items that reflect the concept of interest and contribute to its evaluation (p 12) [8]”. At the time of instrument development, the MDASI-BT included patient and caregiver review of items as part of the initial development and validation work [9].

In the evaluation of a PRO measure’s content validity, guidance indicates that determining whether the concepts contained in the PRO are comprehensive and important to patients is critical [8]. Since the original validation of the MDASI-BT, we have reported a qualitative method for concept elucidation, content domain validation, and item generation for disease- and treatment-specific modules of the MDASI [12]. This method can also be used to confirm and modify existing MDASI modules. As stated by the FDA, the addition of “patient interviews, focus groups and qualitative cognitive interviewing ensures understanding and completeness of the concepts contained in the items (p 7) [8]” and initial validation was limited in use of this strategy. The purpose of this study was to identify symptoms through patient-report in qualitative interviews and explore the significance of these symptoms in patients with a glioma from diagnosis, through active treatment and follow-up and to strengthen initial validation of the MDASI-BT to ensure symptom burden experienced is accurately represented within the instrument. The results of the analysis of these qualitative interviews will be compared to the current content of the MDASI-BT to confirm the content validity of the instrument.

## Methods

This study was approved by The University of Texas MD Anderson Institutional Review Board. The study population included adult glioma patients who presented for routine care to the Brain and Spine outpatient clinic at MD Anderson Cancer Center. Patient eligibility included pathologic diagnosis of a glioma: ≥ 18 years of age, ability to speak and read English, ability to give written consent to participate, and determined physically and cognitively capable to complete the research by clinician. Study staff approached eligible patients during their clinical visit and reviewed contents of the informed consent before acquiring signed consent and enrolling the patient into the study. Prior to receiving any treatment and scan-related results, patients participated in a brief interview.

Interviews were completed by two research assistants (A.A, S.P), with over 5 years of experience in clinical research studies, who trained with research staff in the Department of Symptom Research under the guidance of a knowledgeable qualitative researcher. Interviewers received didactic training in the principles and methods of qualitative research, followed by observation (interviewers watched several patient interviews exploring steps to initiate interview questions and when to utilize probe questions to obtain complete information) and field practice (research staff observed the interviewers several times and concluded with a debriefing period after each interview to provide feedback and concerns), before beginning interviews of glioma patients. Research staff ended the training when confident in interviewers ability to proceed independently.

After patients were consented, qualitative interviews were conducted with the patient in an exam room as they awaited being seen by their physician. The interviewer was required to notify the patient of recording and it was not turned on until patient gave a verbal consent to continue. A list of open-ended questions guided the interview, with an initial question of, “Would you share what your experience has been leading up to today?” If the interviewer identified incomplete information from the patients’ responses, probe questions about changes in health prior to diagnosis, effects of treatment, symptoms at recurrence, factors impacting symptoms, and the overall effects of symptoms on daily living were asked, to clarify the full extent of the patients’ experiences. The interview concluded with the interviewer asking patients for any supplementary information they wanted to share about their diagnosis and treatment experience. All interviews were digitally recorded with patient consent. Average time for interviews was 9 min and 33 s. A field note describing the circumstances of the interview was completed by the interviewer after the interview was completed. Patient interviews and field notes were transcribed verbatim by professional transcriptionists. Following each interview, patients completed a paper copy of the MDASI-BT independently. In rare cases, if the patient had physical difficulties with filling in responses, the interviewer helped with survey completion, recording patient’s independent response on the instrument. Symptoms reported 5 or higher and interference items rated 2 or higher were categorized as moderate to severe. Demographic characteristics on gender, education, employment status, ethnic/racial group was completed following completion of the instrument. Clinical information on disease status and recurrence was completed by the clinician. Interviews were continued until concurrent analysis showed that saturation was reached (no new information was identified in subsequent interviews).

### Subject recruitment

Table 1 presents patient characteristics at time of interview. A sampling grid showing percentage of patients proposed for enrollment was utilized for purposive sampling to assure that adequate numbers of patients with characteristics that might influence the experience of interest, in this case symptoms, were interviewed. Variables within the sampling grid included: gender, age, treatment group (newly diagnosed, on-treatment, active follow-up with no current treatment), race/ethnicity, and current and overall treatment type (radiation, chemotherapy, radiation and chemotherapy).

**Table 1:** Sample Characteristics

	*n* = 23	%
Gender		
Male	11	48%
Female	12	52%
Race		
Black or African American	2	9%
White	19	83%
Asian	1	4%
American Indian/ Alaskan Native	1	4%
Age		
Range	23–62	
Median	53	
Education		
High school	7	32%
Associate degree	2	9%
College graduate	5	23%
Post-graduate	3	14%
Other	5	23%
Employment Status		
Full-time	6	32%
Part-time	3	16%
Retired	3	16%
Unemployed due to dx	6	32%
Unemployed prior to dx	1	5%
Diagnosis		
Astrocytoma	5	22%
GBM	11	48%
Oligodendroglioma	7	30%
Current Imaging results		
Postop Newly Diagnosed	3	13%
Stable	16	70%
Progression	3	13%
Postop Progression	1	4%
History of Recurrence		
No	12	52%
Yes (first time)	5	22%
Yes (repeated)	6	26%
Treatment Group		
Newly Diagnosed	2	9%
On-treatment	13	57%
Follow-up	8	35%
^a^KPS		
70–80	5	22%
90–100	18	79%

*N* = 23

^a^Treatment Groups KPS rating: Newly Diagnosed: 90 = 2(100%), On-treatment: 70–80 = 5 (39%), 90–100 = 8 (62%), Follow-up 90–100 = 8 (100%)

### Analysis

Three authors were involved in the analysis. Each person independently reviewed transcripts, identified symptoms, and overall themes. Authors came together as a group to discuss symptom findings and compile a list of agreed upon symptom codes to utilize throughout analysis of interview data. A thematic approach was used for analysis, focusing on identifying, analyzing and reporting of patterns (themes) [13]. We started with identifying themes within the transcripts on symptoms reported and symptoms occurring together throughout the trajectory of the patient’s illness. Additionally, identifying if symptoms reported during the interviews coincided with items within the MDASI-BT instrument followed. Qualitative analysis was conducted using MAXQDA 2018 (VERBI Software, 2017, [14]) to identify symptom code frequencies per patient and between the different stages throughout the illness trajectory. IBM SPSS Statistics for Mac Version 24 [15] was used for descriptive statistics on the sample characteristics and MDASI findings.

Computer assisted software (MAXQDA 2018) was used to establish symptom codes patients experienced. A code was formed to match each symptom. During the coding process, codes were applied to patient statements within the transcripts corresponding to the respective symptom. For example, a patients’ statement of feeling, “mainly nauseated”, was coded as ‘nausea’. Each symptom was counted once for each patient that reported its occurrence. Attempts were made to explore all symptoms within each patient transcript to identify independent symptom reports and occurrence of any MDASI-BT symptoms. We tracked repeated symptom codes within each transcript but counted the symptom only once for each patient. Additionally, we identified how many of the 22 symptom items from the MDASI-BT each patient reported during the interview. Following the coding process, transcripts were re-read to check if any symptoms were missed. Cases with words or sentences comparable to MDASI-BT symptoms were discussed with the research team before applying the corresponding code. For example, “headaches”, “foot cramps”, “hormonal migraines” and “pain” were coded as “pain” in keeping with the findings from Armstrong et al. [9].The same process used for symptom coding was applied in identifying the symptoms corresponding to the MDASI-BT interference items within the interviews in addition to identifying any additional patient descriptions of interference.

Lastly, we identified symptoms experienced through the disease trajectory, analyzing various stages by expanding our variable list in the sampling grid to include: pre-surgery, after-surgery, during- treatment, after-treatment, and recurrence to which symptom codes could correspond.

## Results

### Demographic characteristics

Table 1 provides the demographic and clinical characteristics of the sample. All patients who were approached agreed to participate in this study. With 23 patients enrolled, saturation in the data was reached, as patients’ report of their experience became repetitious and no new information was being disclosed. The sample was primarily white (83%), with a mean age of 49 (median = 53) and a Karnofsky performance status ≥90 (79. The sample consisted of patients with glial tumors, with 48% having a diagnosis of glioblastoma. The majority of patients were on active treatment (57%), and 48% had experienced one or more recurrences.

### Qualitative interview results

Table 2 shows symptoms of the MDASI-BT that were reported in the interviews. Table 3 provides narrative examples of patient descriptions of those symptoms. Throughout the illness trajectory, patients reported that symptoms (e.g., “short-term memory”, “not getting words out”, “hunger”, “headaches”, “nausea”, “fatigue”, and “not able to think”) impacted their overall disease experience. Overall, twenty of the symptoms reported within the interview are included in the MDASI-BT. On average, patients reported 4 symptoms (median = 4, range 0–9). Overall, the most common symptoms coded for the sample included fatigue (55%), seizures (50%), and pain (50%). The sample on average also reported at least 1 item from the MDASI-BT interference scale during the interview, with interference with work identified by over half (56%), followed by enjoyment of life (44%) and the remainder being reported by at least 27% of the sample.

**Table 2:** Frequency Table of Symptom Codes Compared to MDASI-BT Items

Symptom^b^	Frequency^a^	Percentage
Fatigue	12	55%
Seizures	11	50%
Pain	11	50%
Nausea	7	32%
Remembering things	5	23%
Change in appearance	5	23%
Difficulty speaking	5	23%
Difficulty understanding	4	18%
Vision	4	18%
Weakness on one side	4	18%
Disturbed sleep	3	14%
Irritability	3	14%
Numbness or tingling	3	14%
Drowsy (sleepy)	2	9%
Change in bowel pattern	2	9%
Difficulty concentrating	2	9%
Vomiting	2	9%
Lack of appetite	1	5%
Feeling sad	1	5%
Distressed(upset)	1	5%
Dry mouth	0	0%
Shortness of breath	0	0%
Interference^c^	Frequency^a^	Percentage
Work	9	56%
Enjoyment of Life	7	44%
Walking	5	31%
Relations with other people	4	25%
Mood	4	25%
General Activity	4	25%

^a^Number of patients who endorsed symptom/interference item

^b^Total number of patients that reported a symptom from MDASI-BT = 22

^c^Total number of patients that reported an interference item = 16

**Table 3:** Interview Response Narrative Examples from Most Common Symptoms and Impact

Most Common Symptoms	Patient Response
Fatigue (55%)	“It was terrible, I was just really tired all the time.”
Seizures (50%)	“The seizure is what triggered everything—started everything.”
Pain (50%)	“I had a really bad pain in my neck, back right side—just felt like there’s a lot of pressure on my neck.”
Impact of Symptoms	
Work (56%)	“Well, it affected my work life. At the time, I had the memory problems, I had the GI problem, and we were transitioning from a chairman in our department to a new one. And I needed to be on my toes. I needed to have a good memory. I needed to be there. And that was very, very difficult. So, it put a lot of stress on me because I knew I wasn’t able to be there.”
Enjoyment of Life (44%)	“I was unable to play with my kids as much as usual. I’m usually a very vibrant person, and it took away from that. I wasn’t able to be myself.”
Group Response Examples	
Pre-surgery	“So, I had grand mal seizures—five of them before I was diagnosed correctly.”
After-surgery	“I’ve gotten some lesser sensation on my left side, so sometimes I drag my left leg and sometimes I kind of walk into walls.”
During treatment	“The chemo does you so bad. You’re so tired. You’re so fatigued. It just has you so tired.”
After treatment	“And we knew something was going on, obviously, because I had some seizures before that.”

During qualitative interviews, patients described the symptoms that occurred prior to diagnosis and surgery, during initial treatment, and if applicable, at the time of recurrence, completion of treatment and/or in active follow-up. When looking across the disease trajectory, the most commonly reported symptoms varied (see Table 4). During analysis of the collected data evaluating symptoms experienced throughout the disease course, the majority of coded symptoms were reported as occurring during treatment, highlighting the added treatment associated symptoms. After treatment was completed, symptoms endorsed during this time were limited, with only one patient indicating any problematic issues, while more symptoms were found to be reported in other stages of the illness trajectory. Table 4 lists some symptoms reported that are not included in the MDASI-BT. For additional symptoms not included in the MDASI-BT, decisions were made by the researchers doing the analysis (review of transcripts and reporting) that some symptom codes represented the same symptoms and could be incorporated into existing items. The interview report of running into things, difficulty with balance, and paralysis were included in the interference item walking, mention of foot cramps was included in the symptom item pain, and neuropathy was added to the numbness and tingling symptom item. Additional reported symptoms that are not currently included in the MDASI-BT include tremors, reported by only one patient, swelling and itching, reported by only two patients each. No other symptoms not included in the MDASI-BT were reported by more than one patient.

**Table 4:** Endorsed Symptoms During Interviews at Different Stages of Patient Diagnosis

Symptoms reported	N (%)
Pre-surgery^b^	
Seizures	10 (45%)
Pain	8 (36%)
Difficulty Speaking	3 (14%)
Night sweats^a^	1 (9%)
Hand tremors^a^	1 (9%)
Tongue spasms^a^	1 (9%)
After surgery^b^	
Weakness	2 (25%)
Pain	2 (25%)
Difficulty sleeping	2 (25%)
Noise sensitivity^a^	1 (13%)
Tremors^a^	1 (13%)
Food cravings^a^	1 (13%)
During Treatment^b^	
Fatigue	8 (47%)
Hair loss	6 (35%)
Nausea	6 (35%)
Itching^a^	2 (12%)
Swelling^a^	2 (12%)
Muscles aches^a^	1 (6%)
After Treatment^b^	
Numbness/tingling	1 (100%)
Foot cramps^a^	1 (100%)
Difficulty stretching^a^	1 (100%)
Recurrence^b^	
Numbness	1 (50%)
Walking	1 (50%)
Seizures	1 (50%)

^a^Symptoms not represented in the MDASI-BT

^b^ For each stage, the following represents, the total number of MDASI(M)/NON MDASI (NM) symptoms reported and symptoms not included in the table: *Pre-surgery* (11 M, memory issues, nausea, difficulty understanding, difficulty walking, difficulty concentrating, fatigue, vision, numbness, 7NM: dizziness, withdrawing, difficulty moving body parts, crushing in throat), *After-surgery* (9 M, difficulty understanding, irritability, difficulty walking, numbness, vision, memory issues, 4NM, sneezing changes), *During Treatment* (14 M, pain, unable to speak, mood changes, difficulty walking, weakness, numbness/tingling, vision, change in bowel pattern, difficulty remembering, difficulty concentrating, memory issues, 8NM, decreased sex drive, crustiness on lips, increased appetite, weakness of voice, shakiness), *After Treatment* (1 M, 2NM), *Recurrence* (3 M, 0NM)

#### Reported symptoms prior to and after surgery

The majority of patients (96%) endorsed at least one symptom prior to initial surgery, with seizures (45%), pain (36%), difficulty speaking (14%), and memory issues (14%) being the most prevalent. One patient reported, “I had grand mal seizures - five of them - before I was diagnosed correctly.” At the time of diagnosis but prior to surgery, 18 symptoms were reported with 7 symptoms not specifically represented within the MDASI-BT symptom list (night sweats, hand tremors, tongue spasms, dizziness, withdrawing, difficulty moving body parts, crushing in throat). Three symptoms included in the MDASI-BT (change in appearance, disturbed sleep, weakness on one side), were not reported by patients as occurring prior to surgery. After surgery, 13 total symptoms were reported, with 4 symptoms (noise sensitivity, tremor, sneezing changes, food cravings) not represented in the MDASI-BT.

#### Reported symptoms during treatment

The majority of patients (57%) were receiving treatment at the time of their interview. Seventy-four percent reported an occurrence of at least one symptom during their treatment course. Patients commonly experienced fatigue (47%), change in appearance/distress related to hair loss (35%), nausea (35%), and pain (29%) during treatment. Symptoms reported during treatment included 14 items from the MDASI-BT and an additional 8 symptoms not represented within the instrument (increased appetite, itching, swelling, crustiness on lips, muscles aches, weakness of voice, shakiness, decreased sex drive). Surprisingly, there were 3 symptoms contained in the MDASI-BT that were not reported by patients discussing their treatment experiences (lack of appetite, difficulty speaking (finding the words), and feeling drowsy). For those currently receiving treatment, fatigue was the most common symptom code (69%), with one patient expressing, “I feel more tired now,” due to a prolonged period of taking chemotherapy. Pain symptoms (46%) followed in frequency as a patient expressed, “having headaches everyday”. Problems with remembering things (39%), seizures (39%) and change in appearance (39%) rounded out the top 5 MDASI-BT symptoms endorsed within the instrument.

#### Reported symptoms when on disease surveillance

Overall, ten symptoms from the MDASI-BT were reported by patients in follow-up without active treatment. The most common symptoms reported were seizures (71%), nausea (57%), and pain (57%). This group did not report experiencing the following MDASI-BT symptoms: change in appearance, vomiting, feeling sad, dry mouth, drowsy, lack of appetite, remembering things, shortness of breath, vision, difficulty concentrating, difficulty understanding, and change in bowel pattern. The majority reported ongoing issues with seizures, with one patient describing, “I have attacks that feel similar to anxiety”.

### MDASI-BT questionnaire results

The MDASI-BT was completed by all participants and as outlined in Table 5, twenty of the twenty-two symptoms were endorsed as present in the last 24 h (seizures and vomiting were not endorsed as present in the last 24 h). Upon completing the MDASI-BT, patients reported on average 6.8 symptoms (median = 7) with 14% of the reported symptoms (mean = 3) as moderate to severe (rated ≥5). Common symptoms reported include: fatigue (70%), drowsiness (57%), problems remembering things (57%) and disturbed sleep (57%). Common moderate to severe symptoms reported include: disturbed sleep (35%), drowsiness (30%), dry mouth (22%), weakness (22%), distress (22%), and fatigue (22%). Impact of symptoms on general activity and mood was reported by 45% of participants, and the most severe impact of symptoms reported occurred with work (27% moderate-severe and mean 1.9, SD 2.9). Patients on average reported 2.2 interference items (median 2.0).

**Table 5:** MDASI-BT Instrument Scores and Severity

MDASI-BT	Mean (SD) Range	None	Mild (Scores 1–4)	Moderate-Severe (Scores 5–10)
Symptoms				
Pain	0.9 (2.1) 0–7	17	3	3
Fatigue	2.8 (3.1) 0–10	7	11	5^a^
Nausea	0.6 (1.8) 0–8	19	3	1
Disturbed Sleep	3.0 (3.4) 0–10	10	5	8^a^
Distressed	2.3 (3.2) 0–10	11	7	5^a^
Shortness of breath	0.4 (1.3) 0–6	21	1	1
Remembering things	2.1 (3.1) 0–10	10	9	4
Lack of appetite	1.2 (2.4) 0–8	17	3	3
Drowsy	3.0 (3.5) 0–10	10	6	7
Dry mouth	2.0 (3.1) 0–10	14	4	5^a^
Sad	1.6 (2.4) 0–8	13	6	4
Vomiting	0.0 (0.0) 0	23	0	0
Numbness	1.2 (2.7) 0–9	18	2	3
Weakness	2.1 (3.1) 0–10	13	5	5^a^
Understanding	1.0 (2.5) 0–10	18	2	2
Difficulty Speaking	0.3 (0.8) 0–3	19	4	0
Seizures	0.0 (0.0) 0–0	23	0	0
Difficulty Concentrating	1.5 (2.2) 0–8	13	7	3
Vision	1.2 (2.5) 0–8	18	1	4
Change in Appearance	0.8 (2.6) 0–10	20	1	2
Bowel Pattern	0.8 (2.3) 0–8	20	1	2
Irritability	1.7 (3.0) 0–10	14	6	3
Interference Items				
General Activity	1.8 (3.0) 2–10	13	7	3
Mood	2.0 (3.1) 2–10	13	6	4
Work	2.0 (2.9) 2–10	14	3	6
Relations with other people	1.0 (2.3) 2–10	17	4	2
Walking	1.7 (3.0) 2–10	16	3	4
Enjoyment of life	1.7 (3.1) 2–10	15	4	4

^a^Commonly reported moderate-severe symptoms items on the MDASI-BT

## Discussion

Interviewing patients to understand the scope of symptoms experienced throughout their illness is important to ensure that instruments targeting the patient’s disease encompassed their concerns. Overall the mean symptom severity report on the MDASI-BT in this study was low, but the symptoms were reported across the range of severity and this is not dissimilar to other reports of symptoms in patients with brain tumors [3].

In comparing symptoms reported in the qualitative interview to those included in the MDASI-BT, all symptoms were reported by patients during the interviews with the exception of two (dry mouth and shortness of breath). In previous reports, dry mouth has been shown to be associated with poor performance status [10, 16]. Additionally, dry mouth was a common symptom reported as moderate to severe (22%) within the MDASI-BT instrument completed at the time of the interview. This may reflect that patients experience the symptom but don’t endorse it as being related to their tumor.

Patients reported multiple symptoms throughout the disease trajectory. In the qualitative interviews, fatigue, seizures, and pain were the most commonly reported symptoms occurring overall, but the most common symptoms varied dependent on where the patient was in their disease course. Seizures were commonly reported prior to surgery and also by those who were in follow-up after completing therapy; whereas patients reported treatment-associated symptoms, such as fatigue and nausea during treatment. This finding highlights the importance of treatment-associated symptoms. These findings are similar to previous reports that indicate that symptoms common across cancers also occur in this patient population [3].

A few symptoms (problems with balance, dizziness, tremors, tongue spasms, itching, night sweats) were identified in the qualitative interviews that are not included in the 22 MDASI-BT symptoms. Tremors were included as an initial item during the initial development of the MDASI instrument but did not receive the necessary endorsement by 80% of the review panel for inclusion in the final list of items as being commonly associated with tumors in the adult brain tumor population, but more commonly related to use of corticosteroids. Balance was suggested at that time as an additional item, but expert reviewers concluded the ‘difficulty walking’ item would include difficulties with balance. The identification of these symptoms from the qualitative interviews suggest that there may be additional symptoms to consider in this population, but not to include in a general symptom measure for patients with brain tumors as less than 20% of the sample reported them [17]. Some of these reported symptoms were in the context of concurrent medications and highlight the importance of being cognizant of treatment-associated symptoms. The addition of items as new therapeutic approaches is added to the armamentarium should be considered and included in the existing measure if they become standard treatments in this population. The Department of Symptom Research at MD Anderson provides a symptom library of individual symptoms that can be added to the MDASI-BT module to create an instrument that is tailored to specific research needs. Of note, items are added to the end of the original questionnaire to maintain its integrity and psychometric validity.

As has been reported in previous studies [3, 18], patients endorsed that symptoms interfered with their activity and mood during the interviews and affected aspects of daily living as observed through endorsed interference items within the MDASI-BT. Patients primarily reported impact on general activity, but work and mood were also reported to be impacted by the occurrence of symptoms. The interference items of the MDASI-BT have been shown to be associated with disease progression [18], and survival outcomes [19, 20] and these results continue to support the utility of this report in understanding the clinical impact of symptoms in this patient population.

Symptoms reported independently by patients prior to initial diagnosis coincided with results from The Jumpstart Brain Tumor Drug Development Coalition and FDA Clinical Trials Clinical Outcome Assessment Endpoints Workshop [6]. Weakness and change in bowel pattern were the only two symptoms from the workshop that were not endorsed by our sample. Of the top symptoms (headaches (57%), seizures (40%), changes in speech (26%), changes in mood/personality (26%)) that led to initial diagnosis from survey participants, our sample endorsed similar issues with seizures (45%) as the most common, followed by pain (36%), difficulty speaking (14%) and memory issues (14%). These findings provide insight into the content validity of the MDASI-BT instrument as the symptoms reflected are common symptoms patients experience. Additionally, the results from the workshop found that symptoms patients consider as important factors to clinical assessment [6], including retaining brain functioning (30%), maintaining ability to walk (28%), improving memory or ability to concentrate (13%) and reducing fatigue (5%), coincide with symptom items within the MDASI-BT and can be seen as problematic issues in our patient population. The results show the importance of these symptoms to patients and the need to assess the severity of these issues through the disease trajectory.

As stated in the literature [6], several PROs have the tendency to measure the same symptom in different ways and the MDASI-BT simplifies the process by encapsulating the patient experience into 22 symptoms and 6 interference items. With the ability to be used with other instruments and frequent assessment of the occurrence of symptoms, the MDASI-BT is an important instrument for clinical care practices [10] (Additional file 1).

Limitations to this study include a patient population that can have trouble with recall issues due to their disease or treatment and this may have limited their ability to describe past experiences. The sample did, however, include patients at several touchpoints in the disease trajectory, with the exception of pre-surgery. Patients at the end of life were not included, so symptoms common during that component of the disease trajectory were not assessed. Also, not asking additional questions after the patient had completed the MDASI-BT to clarify any discrepancies between symptom reports within the interviews and the MDASI-BT leaves researchers with an uncertainty on reasons some symptoms were more apparent in the instrument than the interviews. Most of our sample was currently on treatment (57%) and the insignificance of symptoms not reported during the interviews could be influenced by this not impacting various aspects of their life prospectively and/or retrospectively, limiting report of possible key information during the interviews. Lastly, only having a small number of patients exhibiting low KPS, we might have missed additional symptoms experienced throughout the illness trajectory that this group could have added to the findings.

During the initial development of the instrument, items based on review of the literature were reviewed by an expert panel (consisting of expert clinicians and allied health providers as well as patients and caregivers) and a content validity index score was calculated, for each item [9]. At that time the expert reviewers could also suggest other items to include in the instrument. Qualitative interviews were not used in the MDASI-BT validation study, but findings show how relevant the core symptoms established applied to our sample. Our findings show over half (54%) of the 13 core symptoms represented throughout different treatment stages strengthening the use of this instrument to identify relevant symptoms plaguing patients during the illness trajectory. Use of qualitative interviews during the developmental stage of instrument formation to understand the patient experience to help with creation of a holistic measurement to capture patient concerns only validates an instrument more and should be considered as an additional source during the inclusion process of items.

## Conclusions

The findings of this study support the variability in the individual patient symptom experience and need for the inclusion of the MDASI-BT. Furthermore, the results underscore the importance of establishing an item bank to capture specific symptoms experienced throughout the illness trajectory. This can be pivotal in identifying symptoms impacting patients and helps with providing best care practices for this patient population. Using a standardized instrument for such a complex population helps in bridging the gap between unidentified symptoms that interviews inadvertently may not capture. The average number of symptoms present similarly varied between this mixed method approach with more symptoms present from the self-reported instrument than when open-ended questions were asked.

The impact of symptoms throughout the disease trajectory highlights the need in identifying problematic areas that can impede overall quality of life for patients with a glioma. Patients experience debilitating functional impairment from symptoms of their tumor, treatment, and surgery [21]. This instrument allows for repeated measurement of symptoms and the reported interference of symptoms with function (walking, working, and general activity) as well as mood and relationships with others, is integral to management of overall life quality [10].

Applying PRO measures to studies provides important additional information to help evaluate the impact of treatment on patients [22]. Focusing on symptom management through periodic reassessment of symptoms in clinical care should also be emphasized to help ameliorate patient distress and promote well-being throughout the cancer trajectory [10, 21]. This report supports that the MDASI-BT contains a core set of symptoms common to patients with gliomas and may be a useful screening tool in clinical care and measurement for longitudinal evaluation in clinical trials [10].

## Additional file

Additional file 1: The MD Anderson Symptom Inventory for brain tumor (MDASI-BT). (PDF 79 kb)

## Data Availability

All data generated or analyzed during this study are included in this published article.

## Abbreviations

CNS: Central nervous system
COA: Clinical Outcome Assessment
FDA: Food and Drug Administration
MDASI-BT: MD Anderson Symptom Inventory-Brain Tumor
