# Supplementary material for: Identifying symptom recurrences in primary brain tumor patients using the MDASI-BT and qualitative interviews
Source: J Patient Rep Outcomes. 2019 Aug 23;3:58. doi: 10.1186/s41687-019-0143-0 (PMC6708028; doi:10.1186/s41687-019-0143-0)
Supplement: Supplementary file 1 — The MD Anderson Symptom Inventory for brain tumor (MDASI-BT). (PDF 79 kb) [file 41687_2019_143_MOESM1_ESM.pdf]

Date: __ __ / __ __ / __ __
(month) (day) (year)

Subject Initials: __ __ __

MD Anderson # : __ __ __ __ __ __

Study Name: ____________________

Protocol #: ______________________

PI: ___________________________

PDMS # : __ __ __ __

M. D. Anderson Symptom Inventory - Brain Tumor (MDASI - BT)

Part I. How severe are your symptoms?

People with cancer frequently have symptoms that are caused by their disease or by their treatment. We ask you to rate how severe the following symptoms have been in the last 24 hours. Please fill in the circle below from 0 (symptom has not been present) to 10 (the symptom was as bad as you can imagine it could be) for each item.

	Not Present 0	1	2	3	4	5	6	7	8	9	As Bad As You Can Imagine 10
1. Your **pain** at its WORST?	○	○	○	○	○	○	○	○	○	○	○
2. Your **fatigue (tiredness)** at its WORST?	○	○	○	○	○	○	○	○	○	○	○
3. Your **nausea** at its WORST?	○	○	○	○	○	○	○	○	○	○	○
4. Your **disturbed sleep** at its WORST?	○	○	○	○	○	○	○	○	○	○	○
5. Your feeling of being **distressed (upset)** at its WORST?	○	○	○	○	○	○	○	○	○	○	○
6. Your **shortness of breath** at its WORST?	○	○	○	○	○	○	○	○	○	○	○
7. Your problem with **remembering things** at its WORST?	○	○	○	○	○	○	○	○	○	○	○
8. Your problem with **lack of appetite** at its WORST?	○	○	○	○	○	○	○	○	○	○	○
9. Your feeling **drowsy (sleepy)** at its WORST?	○	○	○	○	○	○	○	○	○	○	○
10. Your having a **dry mouth** at its WORST?	○	○	○	○	○	○	○	○	○	○	○
11. Your feeling **sad** at its WORST?	○	○	○	○	○	○	○	○	○	○	○
12. Your **vomiting** at its WORST?	○	○	○	○	○	○	○	○	○	○	○
13. Your **numbness or tingling** at its WORST?	○	○	○	○	○	○	○	○	○	○	○
14. Your **weakness** on one side of the body at its WORST?	○	○	○	○	○	○	○	○	○	○	○
15. Your difficulty **understanding** at its WORST?	○	○	○	○	○	○	○	○	○	○	○
16. Your difficulty **speaking** (finding the words) at its WORST?	○	○	○	○	○	○	○	○	○	○	○

SAMPLE

Copyright 2000 The University of Texas M. D. Anderson Cancer Center
All rights reserved.

Date: ☐☐ / ☐☐ / ☐☐
(month) (day) (year)

Subject Initials: ____________

MD Anderson # : ☐☐☐☐☐☐

Study Name: ____________________

Protocol #: ____________________

PI: ____________________

PDMS # : ☐☐☐☐

	Not Present 0	1	2	3	4	5	6	7	8	9	As Bad As You Can Imagine 10
17. Your **seizures** at its WORST?	○	○	○	○	○	○	○	○	○	○	○
18. Your difficulty **concentrating** at its WORST?	○	○	○	○	○	○	○	○	○	○	○
19. Your **vision** at its WORST?	○	○	○	○	○	○	○	○	○	○	○
20. Your change in **appearance** at its WORST?	○	○	○	○	○	○	○	○	○	○	○
21. Your change in **bowel pattern** (diarrhea or constipation) at its WORST?	○	○	○	○	○	○	○	○	○	○	○
22. Your **irritability** at its WORST?	○	○	○	○	○	○	○	○	○	○	○

SAMPLE

Part II. How have your symptoms interfered [illegible]

Symptoms frequently interfere with h[illegible] we [illegible] function. How much have your symptoms interfered with the following items in the last 2[illegible]:

	Did not interfere 0	1	2	3	4	5	6	7	8	9	Interfered Completely 10
23. **General activity?**	○	○	○	○	○	○	○	○	○	○	○
24. **Mood?**	○	○	○	○	○	○	○	○	○	○	○
25. **Work (including work around the house)?**	○	○	○	○	○	○	○	○	○	○	○
26. **Relations with other people?**	○	○	○	○	○	○	○	○	○	○	○
27. **Walking?**	○	○	○	○	○	○	○	○	○	○	○
28. **Enjoyment of life?**	○	○	○	○	○	○	○	○	○	○	○

Copyright 2000 The University of Texas M. D. Anderson Cancer Center
All rights reserved.
